# The role of colonoscopy in the management of intestinal obstruction: a 20-year retrospective study

**DOI:** 10.1186/1471-230X-10-130

**Published:** 2010-11-08

**Authors:** Konstantinos H Katsanos, Mariana Maliouki, Athina Tatsioni, Eleftheria Ignatiadou, Dimitrios K Christodoulou, Michael Fatouros, Epameinondas V Tsianos

**Affiliations:** 1 Department of Internal Medicine & Hepato-Gastroenterology Unit, University Hospital of Ioannina, Greece; 2 Department of Family Medicine, University Hospital of Ioannina, Greece; 3 Department of Surgery, University Hospital of Ioannina, Greece

## Abstract

**Purpose:**

The aim of the study was to assess the use colonoscopy over time in the assessment of large bowel obstruction in a tertiary university hospital.

**Methods:**

Retrospective analysis of surgical and colonoscopy records for the years 1990-2009 in a university hospital. All patients diagnosed with non-conservatively managed bowel obstruction were included.

**Results:**

We recorded 644 patients diagnosed with non-conservatively managed bowel obstruction. Four hundred forty-one (67.3%) were managed only by surgery, 157 (23.6%) were managed by colonoscopy, and 46 (6.9%) by combined colonoscopy and surgery. Patients over 77 years were more likely to receive colonoscopy as monotherapy or combined with surgery as compared to younger patients. Management by colonoscopy only and by combined colonoscopy and surgery increased over time.

**Conclusions:**

Colonoscopy in the management of non-conservatively treated bowel obstruction increased over time. However, therapeutic colonoscopy still has a limited role in bowel obstruction either as monotherapy or combined with surgery.

## Background

Intestinal obstruction represents a severe complication and a potential emergency. Intestinal pseudo-obstruction usually affects the colon but the small intestine may also be involved, and may present in acute, subacute or chronic form [1,2].

Bowel obstruction or pseudo-obstruction can be the result of mechanical causes or motility disturbances [3]. The syndrome of acute colonic pseudo-obstruction is well delineated but its aetiology remains poorly understood [4-7].

Non-operative measures include pharmacologic colonic decompression combined with general measures [8-14] and digestive colonoscopy. Diagnostic colonoscopy in the involvement of the large intestine or enteroscopy in the case of incomplete obstruction of the small intestine is the method indicated in the majority of obstructive intestinal lesions [15]. Fulminant colitis and toxic megacolon represent potential exceptions.

The successful management of intestinal obstruction depends on early diagnosis. A judicious and timely use of both medical and surgical therapies has been proposed for improving outcomes. With a combined multidisciplinary approach, morbidity can be reduced and patients can have a rapid return and improved quality of life [16].

In this study, we aimed to assess what type of treatment (surgical, endoscopic, or both) was used and how much the treatment modalities changed over time among patients with intestinal obstruction in a tertiary university hospital in Northwestern Greece.

## Methods

### Single referral center study

Both departments of endoscopy and surgery are located in the same hospital, have long-term collaboration and are sharing common hospital facilities. The department of internal medicine with its endoscopy unit is a referral center for Gastroenterology and Hepatology and has availability of all endoscopic facilities and treatment modalities for diagnostic, therapeutic and palliative endoscopy. The department of surgery is a referral center for surgery and has availabilities for any type of surgical procedure.

### Retrospective analysis

A retrospective analysis of all surgical records and colonoscopy reports for the years 1990-2009 (first seven months) was performed. All patients diagnosed with small or large bowel obstructions of any type were included. Diagnosis of any type of obstruction was based on patient history, clinical examination and radiological examination.

Surgical, endoscopic or combined management of obstruction was unselected and on individual basis upon treating physician's experience. All patients were treated on hospital basis and no specific protocol from surgeons or internists was followed all these years.

### Surgical, endoscopic or combined management of obstruction

In all patients we recorded data on demographics, clinical characteristics and surgical, endoscopic or combined surgical and endoscopic management. Data on surgical management of obstruction included history of previous surgery, timing of the operation (urgent-scheduled), surgical finding and type of surgical intervention for obstruction.

Data on endoscopic management of obstruction included information on bowel cleansing, end point of colonoscopy, number and type of findings - if more than one - and method, if any, used for the treatment of obstruction. We also recorded whether patients needed more than one colonoscopy. Combined management was characterized any endoscopic procedure performed either pre-operatively or at the operation table.

### Ethical considerations

All patients gave informed consent prior to colonoscopy and surgery and every procedure was according to the rules of good clinical practice. This study was reviewed an approved by our hospital human clinical research ethics committee.

### Statistical analysis

Percentages were calculated for binary and categorical variables while continuous variables were described with median and interquartile range (IQR). We used the SPSS 13.0 (SPSS Inc., Chicago, IL) for the analyses.

## Results

### Obstruction cohort

In total 26,065 surgical records and 18,793 colonoscopy reports were reviewed. We recorded 644 patients with obstruction of any type, which was managed non-conservatively (with surgery alone or colonoscopy alone or with combined surgery and colonoscopy). The demographics and clinical characteristics of the obstruction cohort are presented in Table 1.

**Table 1:** Characteristics both for the total cohort and for each group of bowel obstruction (surgery, endoscopy) separately.

Characteristic	Total (N, %) n = 644	Surgery only (N, %) n = 441	Endoscopy only (N, %) n = 157	Surgery & Endoscopy (N, %) n = 46
Males	363 (100)	230 (65)	96 (27)	28 (8)
Females	292 (100)	211 (73)	61 (21)	18 (6)

Age (median, IQR) years	70 (55-77)	67 (53-76)	72.5 (62-79)	71 (59-79)

Patients previously operated				
Yes	76 (100)	42 (56)	21 (28)	12 (16)
No	579 (100)	399 (70)	136 (24)	34 (6)

Emergency of intervention				
Urgent (within 24 h)	104 (100)	80 (77)	8 (8)	16 (15)
Scheduled	551 (100)	361 (67)	149 (28)	30 (5)

### Obstruction management and outcomes

The characterisitics of surgical and endoscopic management of intestinal obstruction are summarized in Table 2.

**Table 2:** Characteristics for surgical and endoscopic procedures in patients with bowel obstruction.

Parameter	No	%
Bowel cleansing on endoscopy (*n = 203*)		
*Good*	117	58.1
*Moderate*	48	23.3
*Poor*	38	18.6

End point at endoscopy (*n = 203*)		
*Terminal ileum or cecum*	102	48.3
*Ascending colon or transverse colon*	27	18.9
*Descending or sigmoid colon*	30	15.7
*Rectum*	32	16.1
*Endoscopy impossible*	2	1

Endoscopic procedure (*n = 203*)		
*0 = none*	136	64
*1 = endoscopic decompression*	12	6
*2 = tube placement*	4	2
*3 = lesion biopsy*	53	25
*4 = dilatations*	5	2
*5 = volvulus derotation*	1	1

Type of surgical intervention (n = 487)		
*Total or subtotal colectomy*	33	7
*Segmental colectomy*	238	49
*Hartman sigmoidectomy*	37	8
*Left or right hemicolectomy*	123	25
*Ileo-rectal/ileo-transverse anastomosis*	16	3
*Adhesiotomy*	25	5
*Strictureplasty*	11	2
*Foreign body removal*	4	1

Surgical findings (biopsy based, n = 485)		
*Neoplastic*	135	28
*Non-neoplastic*	350	72

In all patients undergoing colonoscopy, bowel cleansing was performed with two to four enemas. One hundred thirty-five (27%) of cases that were operated were diagnosed with neoplasia as the ultimate surgical finding. Sixteen patients had clear evidence of Oglivie's syndrome or but none of them was diagnosed with morbus Hirschsprung. No patient underwent colonoscopy due to immediate postoperative ileus. We had no complications occurring by the use of a stent (perforation or dislocation) and we had not recorded any case of a patient that the colonoscopic intervention could spare a stoma. However, it is only during the last years that colon stenting has become a routine in our endoscopy suites and stenting is a facility not always available and not always feasible during emergent colonoscopy.

Overall, in 102 (48.3%) of patients the scope reached at least the ceacum while colonoscopy was impossible in only 2 patients (1%). In detail 67 out of 203 patients (33%) that were scoped for bowel obstruction underwent the following endoscopic procedures: endoscopic decompression (12), tube placement (4), lesion biopsy (53), dilatations (5) and finally one patient underwent successful sigmoid volvulus derotation. The number of therapeutic endoscopic interventions was 17/203 (8.4%).

Surgical intervention was done by segmental colectomy in 238 (49%) out of the 487 participants who were operated. In addition, 33 patients (7%) underwent total or subtotal colectomy (12 stomas among them), while the remaining 216 patients (44%) underwent other type of surgical procedures including Hartman sigmoidectomy (37), left or right hemicolectomy (123), ileo-rectal or ileo-transverse colon anastomosis (16), adhesiotomy (25), strictureplasty (11) and foreign body removal (4) [Table 2].

### Surgical management

In total 441 (67.3%) patients were managed only by surgery. Using as denominator the total number of patients presenting with non-conservatively (surgery ± colonoscopy) managed obstruction the management of bowel obstruction by using surgery decreased over the years (Table 3).

**Table 3:** Patients presenting with non-conservatively (surgery ± endoscopy) managed obstruction.

	Year quartiles			
	
	≤1997	1998-2002	2003-2005	≥2006
Surgery only (N, %)	135 (95.1)	130 (76.0)	91 (68.9)	85 (42.7)

Endoscopy only (N, %)	7 (4.9)	36 (21.1)	33 (25.0)	81 (40.7)

Surgery and endoscopy (N, %)	0	5 (2.9)	8 (6.1)	33 (16.6)

Total (N, %)	142 (100.0)	171 (100.0)	132 (100.0)	199 (100.0)

### Endoscopic management

In total 157 patients were managed only by colonoscopy. In half of the patients undergoing colonoscopy the colonoscopist was able to reach the terminal ileum or the ceacum. In the elderly patients (>77 years) there was an increase in the use of colonoscopy as monotherapy or combined with surgery in the management of obstruction (Table 4). By contrast, in younger ages (<77 years) the management of obstruction is mainly done by surgery as monotherapy (Table 4).

**Table 4:** Patients presenting with non-conservatively (surgery ± endoscopy) managed obstruction.

	Age quartiles			
	
	<56 years	56-70 years	71-77 years	>77 years
Surgery only (N, %)	115 (29.4)	110 (28.1)	85 (21.7)	81 (20.7)

Endoscopy only (N, %)	23 (16.7)	38 (27.5)	29 (21)	48 (34.8)

Surgery and endoscopy (N, %)	8 (17.8)	14 (31.1)	10 (22.2)	13 (28.9)

Total (N, %)	146 (25.4)	162 (28.2)	124 (21.6)	142 (24.7)

### Combined surgical and endoscopic management

In total, 46 patients were managed by combined surgery and colonoscopy. (Table 3).

## Discussion

This is a retrospective study describing the role of surgical, endoscopic and combined management of intestinal obstruction. This study focused on the role of colonoscopy in suspected large bowel obstruction but not on small bowel obstruction as small bowel enteroscopy and even capsule endoscopy were not available during this 20-year period of our clinical practice.

Surgical management was more likely to be performed in younger patients and patients with small intestinal obstruction. Segmental colectomy was the most frequent type of surgery. Half of the patients who underwent colonoscopy, they also received a colonoscopic intervention. Cancer was the most frequent diagnosis in the patients with obstruction. The study clearly demonstrated the role of colonoscopy and combined surgery with colonoscopy in the management of intestinal obstruction, especially in large bowel intestinal obstruction in the elderly patients.

The use of colonoscopy in the management of bowel obstruction may be consistent with the advantages offered by this approach. First, by reaching endoscopically the terminal ileum or the ceacum, in many patients a possible diagnosis was confirmed or excluded avoiding an unnecessary surgery. It has to be emphasized that reaching the terminal ileum or cecum is not in every case of obstruction necessary (e.g. rectal cancer and ileus). In addition, in almost half of patients scoped an colonoscopic interventional method was applied resulting either in obstruction definite therapy, in surgery-assisting diagnosis, or palliation.

Our study demonstrated the role of colonoscopy as monotherapy or combined with surgery therapy especially in the elderly patients, over 77 years of age. In this age group smooth management of intestinal obstruction is mandatory in order to avoid unnecessary surgery and unexpected complications due to co-morbidities. In the absence of clinical, laboratory or radiological signs of bowel necrosis or perforation, operative and therapeutic colonoscopy is increasing in indications and possibilities. However, experience is needed as complication rates in such procedures are higher compared to routine endoscopies [17].

Our study had several limitations. First, the data were retrospectively collected and analysed. A proper review of management of bowel obstruction would start with hospital diagnosis at discharge or, even before admission, in the department of emergencies. Case selection has excluded all the patients who did not undergo surgery or colonoscopy. This means that interpretation of the importance of the data is limited to hospitalised cases undergoing these two interventions: surgery or/and colonoscopy. However, we tried to conform to the reporting guidelines for observational studies according to the STROBE statement [18]. Second, in our study we could not evaluate the endoscopist's role in the initial assessment of acute intestinal obstruction. In acute intestinal obstruction, the clinician must characterize the level of emergency of the case and distinguish between acute small bowel and acute colonic obstruction [19].

From another point of view the study simply shows an increased use of non-therapeutic colonoscopy over the last five years. However, the proportion of therapeutic procedures is still very low. An alternative explanation is that a lot of patients who were managed conservatively before 2005, now undergo colonoscopy. One could argue that an increased use of colonoscopy does not prove that this approach is helpful. It just may shows that currently the use colonoscopy is more often. Others might argue that a lot of unnecessary colonoscopies may have been undertaken or that there was a replacement of contrast radiology with colonoscopy'.

Additionally, this study did not identify patients undergoing upper gastrointestinal endoscopy for decompression of small bowel obstruction and we are not aware of such studies. May be in the future upper gastrointestinal endoscopy will resolve some obstructions and avoid subsequent surgery. Furthemore, the increasing availability of colonoscopists on urgent basis in many hospitals may facilitate the obstruction management since the surgeons will also use the bowel 'inside' information in addition to the radiology findings [20-25]. As we also observed herein, that some of the cases of large bowel volvulus can be successfully managed only by colonoscopy. However, although emergency endoscopic decompression of the sigmoid volvulus is safe and effective as an initial treatment it may present an early recurrence rate [26]. In pseudo-obstruction, colonoscopy is indicated, [27] with the exception of toxic megacolon [13].

In general, in patients presenting with bowel obstruction colonoscopy has a more limited and selective role compared to surgery and of course for the majority of our patient cases with bowel obstruction surgery was, as expected, the mainstay of therapy. Colonoscopists do colonoscopy - these can be gastroenterologists or surgeons. One third of cases in our series presented either with mild symptoms or had some other characteristics jeopardizing the immediate decision for surgery. In one third of patients undergoing colonoscopy, an intervention was decided and was proved successful in all cases with no complications. In addition, colonoscopy proved to be the definite treatment in 8% of patients scoped who avoided surgery.

As emergent colonoscopy is largely available, in some instances it is logical to attempt a first view of the bowel. In some instances colonoscopists are performing operative colonoscopy, which is assisting surgery. This also explains why the combined management of bowel obstruction increased significantly over the years in our center but we believe also that this also occurs in other centers where colonoscopy on emergency is available.

Since there are no guidelines or algorithms incorporating systematically the use of colonoscopy in the management of bowel obstruction we had to rely upon data, which were based on individual patient approach.

## Conclusions

This study described the long-term experience of a referral center regarding the role of surgery and colonoscopy in intestinal obstruction. Large prospective controlled randomized trials are needed to assess the effectiveness for each modality on the management of bowel obstruction Colonoscopy was increasingly performed over the years in patients presenting with non-conservatively treated bowel obstruction. However, colonoscopy that includes a treatment procedure is still limited for those patients.

## Competing interests

The authors declare that they have no competing interests.

**All authors read and approved the final manuscript**.

## Authors' contributions

KHK implemented the research hypothesis, coded the data and drafted the manuscript

MM collected the data and did the literature search

AT revised the data and performed the statistical analysis

EI assisted in data collection

DKC revised and approved the manuscript

MF revised and approved the manuscript

EVT revised and approved the manuscript

## Pre-publication history

The pre-publication history for this paper can be accessed here:

http://www.biomedcentral.com/1471-230X/10/130/prepub
